# A Wireless sEMG Recording System and Its Application to Muscle Fatigue Detection

**DOI:** 10.3390/s120100489

**Published:** 2012-01-05

**Authors:** Kang-Ming Chang, Shin-Hong Liu, Xuan-Han Wu

**Affiliations:** 1 Department of Photonics and Communication Engineering, Asia University, Taichung 41349, Taiwan; E-Mail: changkm@asia.edu.tw; 2 Graduate Institute of Clinical Medical Science, China Medical University, Taichung 41349, Taiwan; 3 Department of Computer Science and Information Engineering, Chaoyang University of Technology, Taichung 41349, Taiwan; E-Mail: s9827626@cyut.edu.tw

**Keywords:** sEMG, muscle fatigue, Bluetooth, regression slope

## Abstract

Surface electromyography (sEMG) is an important measurement for monitoring exercise and fitness. Because if its high sampling frequency requirement, wireless transmission of sEMG data is a challenge. In this article a wireless sEMG measurement system with a sampling frequency of 2 KHz is developed based upon a MSP 430 microcontroller and Bluetooth transmission. Standard isotonic and isometric muscle contraction are clearly represented in the receiving user interface. Muscle fatigue detection is an important application of sEMG. Traditional muscle fatigue is detected from the median frequency of the sEMG power spectrum. The regression slope of the linear regression of median frequency is an important muscle fatigue index. A more negative slope value represents a higher muscle fatigue condition. To test the system performance, muscle fatigue detection was examined by having subjects run on a pedaled-multifunctional elliptical trainer for approximately 30 minutes at three loading levels. Ten subjects underwent a total of 60 exercise sessions to provide the experimental data. Results showed that the regression slope gradually decreases as expected, and there is a significant gender difference.

## 1. Introduction

Physiological monitoring with wireless transmission can be applied to many applications [1], such as healthcare monitoring with portable devices [2]. Chang *et al.* used a wireless system to monitor sleep action [3]. Yu *et al.* developed a wireless medical sensor measurement system, inclusive of an electrocardiograph, body skin temperature, eye movement detection, electromyography (EMG), motion detection and muscle strength, to detect fatigue in multiple sclerosis patients [4]. Among these physiological measurement systems, EMG is an important non-invasive measurement for monitoring muscle fatigue. EMG can be divided into implantable recording or surface skin recording. Implantable myoelectric sensors have also been developed for intramuscular electromyogram recording [5]. Surface EMG (sEMG) is measured by electrodes attached to the surface of the skin, above the muscle of interest. There are many novel application based upon sEMG, such as upper limb prosthesis control [6], exercise and fitness monitoring [7,8]. sEMG is also used to monitor changes in corticospinal function and ankle motor control during recovery from incomplete spinal cord injury [9].

Muscle fatigue detection is one of the important issues among the applications of EMG. There are a variety of articles discussing muscle fatigue detection by surface EMG (sEMG) amplitude and frequency [10,11] Muscle fatigue is a complicated phenomenon that results from insufficient blood oxygen and nutrition. There are three types of fatigue [12]: (1) central fatigue, (2) fatigue of the neuromuscular junction and (3) muscle fatigue. Muscle fatigue is used of interest in sports medicine, as it gives an estimation of global fatigue of the organism. There are three types of controlled muscle contractions: (1) isotonic (maintain same force), (2) isometric (maintain same position), and (3) isokinetic (maintain same velocity). Isometric contraction is a static movement. The subject is required to be held in a static position; isotonic contraction is a dynamic movement. Local muscle fatigue can be continuously monitored by sEMG, using the maximum isometric and isotonic contraction parameters. It can also demonstrate the biochemical and physiological changes in muscles during fatiguing contractions. The advantages of sEMG are the non-invasiveness and real-time fatigue monitoring during the performance of defined work; it can also monitor the fatigue of a particular muscle that is highly correlated with biochemical and physiological changes in muscles during fatiguing.

Power spectrum analysis is the main EMG signal analysis method. Several spectral sEMG analyses methods can be used to reveal changes in electrophysiological characteristics and, therefore, its validity to assess muscle fatigue [13]. Gonzalez investigated several EMG power spectral indices during dynamic muscle contractions [14]. Spectral parameters such as mean frequency (MNF) and median frequency (MF) are used as fatigue indices during dynamic contractions until exhaustion [14,15]. The MNF and MF always shift to the low frequency when muscle fatigue has occurred. Although muscle fatigue is one of the important applications of sEMG measurement, along with the need for exercise and rehabilitation programs, there are still few sEMG recording systems with appropriate wireless transmission functions. The main problem is the high transmission rate required. In general, the sample frequency for measuring sEMG is above 1 kHz [13–15]. If MNF is used to evaluate muscle fatigue, the sample frequency must be 2 kHz. Therefore, the goal of this study was to develop a wireless sEMG recording system with a 2 kHz sample rate for muscle fatigue estimation.

## 2. Hardware Architecture

**Figure 1.**
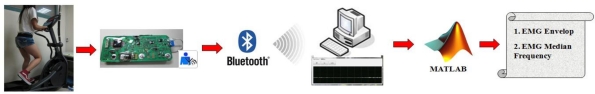
Structure of wireless sEMG recording and muscle fatigue detection and estimation system.

The structure of this wireless EMG recording and muscle fatigue detection system is illustrated in Figure 1. This system is based on the MSP430-F5438 microcontroller as the core structure. The EMG signal is recorded from electrodes attached to the subject and transmitted to the amplifier circuit. The surface electrodes used for the EMG recording were Ag/AgCl 10 mm diameter on self-adhesive supports, and inter-electrode distance was 5 cm. A microcontroller converts the recorded data to a digital signal through a 12-bit analog-to-digital converter (ADC) embedded in the MSP430-F5438. The digital EMG signal is then transferred to a Bluetooth chip and transmitted wirelessly to a remote server. A Visual Basic-based interface system is used to receive the Bluetooth signal and is also used as a real-time signal display and storage. Further EMG signal analysis is performed by a Matlab coded program. Detailed information on each component is provided next.

### 2.1. sEMG Sensor and Amplifier

**Figure 2.**
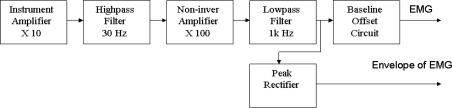
Block diagram of analog circuit for the portable sEMG device.

Figure 2 shows the block diagram of the analog circuit for the portable sEMG device. The raw sEMG signal is a low-amplitude signal; therefore, it needs to be amplified. An instrument amplifier (AD8236, ADI Company), with a gain of 10, is used to enhance the signal. A traditional operational amplifier (AD 8609, ADI Company) is used in the design of a filter, amplifier, peak rectifier, and baseline offset circuit. A two-order Butterworth high-pass filter (cutoff frequency 30 Hz) is used to remove the direct current (DC) offset and baseline wandering, and a two-order Butterworth low-pass filter (cutoff frequency 1 kHz) is used to reduce high frequency noise and to avoid aliasing. The gain of the non-inverting amplifier is 100. The peak rectifier, a parallel circuit of a resister (10 kÙ) and a capacitor (1 μF), is used to extract the envelope of the sEMG as an appropriate measure of change in muscle activity. Finally, the baseline of sEMG signal was raised to 1 V by a baseline offset circuit. The power supply for the measurement system is a 4.5-V lithium battery. A voltage regulator (XC62FP) is used to provide a regulated 3.3 volts for this circuit.

### 2.2. MSP430 Microcontroller

**Figure 3.**
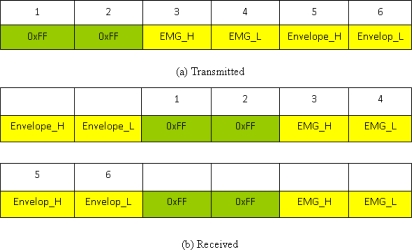
Data transmitting and data receiving modes in the Bluetooth protocol.

The core of the system is the microcontroller (MCU), a MSP430-F5438 chip from Texas Instruments; this is a popular device, especially for biomedical signal acquisition systems [16]. This device has an embedded 16-bit ADC, 256 kB + 512 B flash memory, 16 kB RAM, and needs a voltage of between 1.8 and 3.6 V and a current of between 0.1–250 μA when operated at 24 MHz. A 16-bit timer in the MSP430-F5438 chip was used to enable the ADC 12 to acquire two channel signals, EMG and its envelope. The sampling frequency is 2 kHz. The MCU used the serial communication port (baud rate: 115,200) to connect to the Bluetooth module. Because the A/D converter of the MCU is 12 bits, the sample data were separated into low and high bytes. Therefore, in one sample point, there are two sample data from the sEMG and its envelope which are separated by 4 bytes and stored in a buffer. In this device, when the Bluetooth is suddenly interrupted and communication stopped, some bytes are still being stored in the Bluetooth buffer, and when the Bluetooth transmits data again, these bytes would appear before the new data. Hence, we transmitted 2 bytes, FF and FF, as the distinguishing code before the 4 sample data to distinguish each sample point’s data, as shown in the transmitted form of Figure 3(a). When the Bluetooth model received the data, 6 bytes were considered as a segment. We first found the position of the distinguishing code. Then, one sample point’s data would be acquired between two distinguishing codes, as shown in the received form of Figure 3(b).

### 2.3. Bluetooth Chip

Bluetooth was chosen as the wireless transmission interface in this study due to its low cost and low-power radiofrequency transmission. It is widely used in the biomedical field for functions such as heart beat monitoring and healthcare data transmission. A Bluetooth chip made by the CSR Company, type number BTM-204B, was used. This Bluetooth chip is energy saving and has a high transmission capacity of 1 megabits per second, with pin code. The hopping rate is 1,600 hops per second. It is also easy to integrate into the low-level circuit requirements. The transmission range is limited to within 10 m, and the highest transmission rate is 11 MB. This is still a very powerful wireless transmission tool in terms of the requirements of a home environment.

### 2.4. User Interface

**Figure 4.**
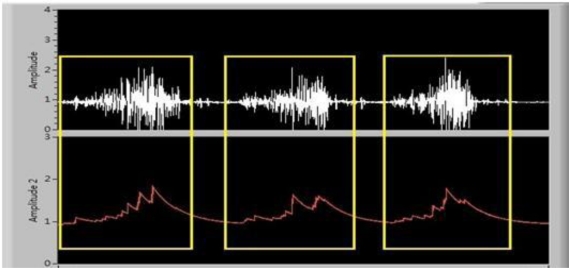
Display of the received EMG signal. The isotonic contraction signal (upper waveform) and corresponding waveform envelope (lower waveform).

A remote personal computer (PC) receives the Bluetooth-transmitted data and connects to the host PC through Universal Telecommunication Radio Access (UTRA). A Visual Basic-based user interface was designed to collect the data package from the Bluetooth device through a serial COM port. Transmitted sEMG and its envelope signals were examined and displayed on a monitor. Offline sEMG signal analysis was performed, and the data was saved in a text file format. Figure 4 is the received sEMG and its envelope signal.

## 3. Hardware Performance Examination

**Figure 5.**
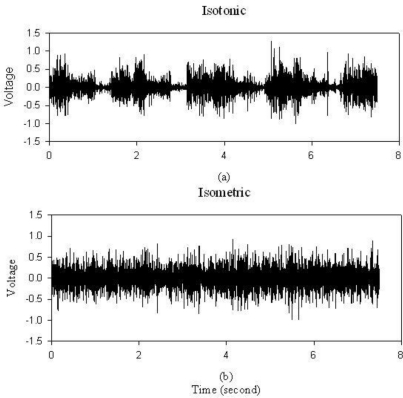
sEMG hardware performance examination for (**a**) 7.5 seconds isotonic contraction. (**b**) 7.5 seconds isometric contraction.

To test the sEMG hardware performance, a dumbbell of 20 pounds was held for one minute. In Figure 5(a), periodic muscle contraction is obvious with this isotonic contraction exercise. Figure 5(b) is the result of isometric contractions. It is obvious that this portable sEMG system is capable of recording muscle contraction effectively.

## 4. Application to Muscle Fatigue Detection

### 4.1. Experiment Procedure

**Figure 6.**
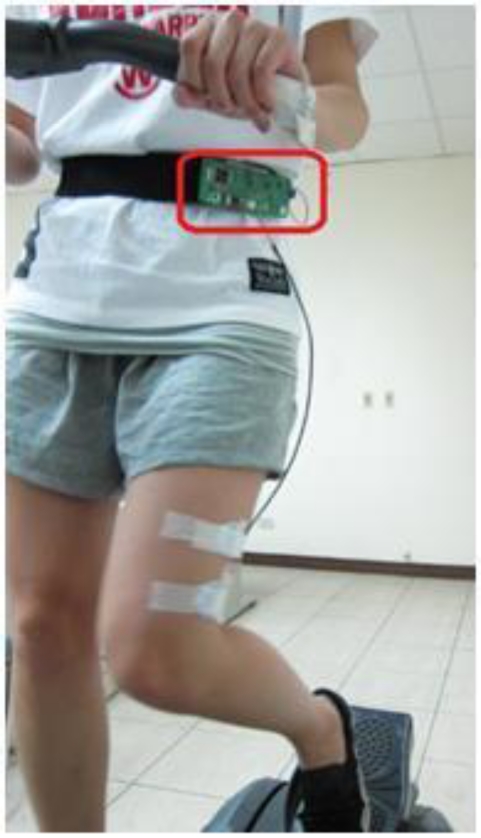
Illustration of wireless sEMG device worn on the lateral waist with electrodes placed on the left vastus lateralis.

There were ten volunteers involved (five male, and five female), with ages ranging from 19 to 27 years. Their information is listed in Table 1. Before data collection, a consent form was signed by each subject. The developed wireless sEMG device is worn on the lateral waist. The upper edge of left patella was used as the reference point. Electrodes are placed at 15 cm position above the reference point, and close to the medium of left vastus lateralis, as shown in Figure 6. The sEMG data is transmitted to the host computer and recorded. The subjects were required to run on the pedaled-multifunctional elliptical trainer (Johnson E8000) for monitoring their muscle fatigue conditions.

An exercise based muscle fatigue examination procedure was established as follows:

Step 1: Subjects wear the wireless sEMG device during the procedure. Alcohol is used to clean the electrode surface prior to smearing electrolytic gel on the electrodes to decrease contact impedance. Athletic tape is used to fix the electrodes and so to avoid movement of the electrodes.
Step 2: There are three load levels in the pedaled-multifunctional elliptical trainer, L2, L4 and L6, with L2 being light and L6 being heavy. The speed of L2 is 55–60 steps per minute (SPM) for males and 50–55 for females. The speed of L4 is 60–70 SPM for males and 56–65 for females. The subjects were required to run at their maximum speed until exhaustion on L6, the speed being greater than that on L4. Ten minutes is the set time for both for L2 and L4.
Step 3: Each subject was recorded twice a week, and there were a total of six recording times for each subject.

### 4.2. Muscle Signal Processing

The recorded sEMG is divided into many segments and a Fast Fourier Transform is performed. The MF of each segment is extracted. MF is defined as the frequency where the accumulated spectrum energy is half of the total spectrum energy, as shown in Equation (1):

(1) $$\int_{0}^{\mathrm{MF}}\;P\left(f\right)\mathrm{df}=\int_{\mathrm{MF}}^{\infty }\;P\left(f\right)\mathrm{df}=\frac{1}{2}\;\int_{0}^{\infty }\;P\left(f\right)\mathrm{df}$$

where p(f) is the power spectrum density of sEMG.

**Figure 7.**
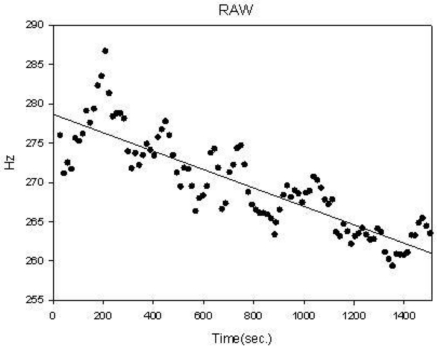
A typical illustration of median frequency distribution and regression line for one subject for 30 minutes exercise.

The window size of the sEMG segment is 30 seconds, and step size is 15 seconds. In order to quantify the distribution of MF during the examination of three stages, a linear regression analysis was applied to evaluate the muscle fatigue condition [17]. The linear function is defined as:

(2) y=Ax+b

where *y* is MF and *x* is the time interval, *A* is the regression slope and *b* is the bias. The greater the muscle fatigue, the smaller the slope [17]. We also used the correlation coefficient (*r*) to represent the stability of sEMG in terms of muscle fatigue, which is defined as:

(3) $$r=\frac{\sum_{i=1}^{n}\left(x_{i}-\bar{x}\right)\;\left(y_{i}-\bar{y}\right)}{\sqrt{\sum_{i=1}^{n}\;{\left(x_{i}-\bar{x}\right)}^{2}\;\sqrt{\sum_{i=1}^{n}\;{\left(y_{i}-\bar{y}\right)}^{2}}}}$$

where *x̄* and *ȳ* are are average of *x* and *y*, respectively. In this study, *x* is time and *y* is MF frequency. The more stable sEMG, the larger the *r* value is. A typical MF distribution is shown in Figure 7. It is well known that MF will decrease as muscle exercise time increases. Parameter r and A will be used to describe the variability and degree of muscle fatigue.

### 4.3. Statistics

In this study, the SIGMAPLOT software package was used for data analysis. Descriptive statistics were applied to subjects’ personal information and muscle fatigue parameters (regression slope and correlation coefficient). Data were represented as mean ± standard deviation (mean ± SD). Statistical testing between male and female on muscle fatigue parameters was examined by t-test. Significance test for the alpha value was set at 0.05.

## 5. Results and Discussions

The MF regression parameters distribution for 30 minutes of exercise is shown in Table 2.

There is a significant gender difference for muscle fatigue, as seen in the regression slope during L4 (male −0.0088 ± 0.0070, female −0.0161 ± 0.0113, p < 0.01 **) and L6 (male −0.0128 ± 0.0095, female −0.0257 ± 0.0175, p < 0.001 ***), and also during L2 (male 0.6079 ± 0.2366, female 0.4192 ± 0.2774, p < 0.01 **), L4 (male 0.4912 ± 0.2259, female 0.6383 ± 0.2196, p < 0.05 *) and L6 (male 0.5080 ± 0.2220, female 0.6393 ± 0.2729, p < 0.05 *) load level with correlation coefficient parameter. There is no gender difference on both parameters for the whole 30 minute session. A lower slope is an index of higher muscle fatigue. At the beginning of the experiment (L2 session), the slope between gender is very close and there is no statistical difference. During the exercise time, the slope for females is lower than that of males.

Table 3 shows the comparison of different exercise levels for all subjects. There is a significant difference for muscle fatigue shown at L4 and L6 of the regression slope (L4 −0.0125 ± 0.0100, L6 −0.0193 ± 0.0154, p < 0.01 **). Because L6 belongs to the maximum voluntary contraction, then its associated muscle fatigue is more serious than L4. The mean slope of L6 is lower than the mean slope of L4. This result can be a demonstration of the reliability of this wireless sEMG recording system.

With the EMG signal being around 100–1,000 Hz, wireless EMG transmission is not easy to accomplish due to the high frequency components. According to Nyquist’s theory, sampling frequency must be twice as high as the maximum signal frequency. Wireless transmission capacity is the limiting factor for sampling frequency. With a suitable arrangement of MSP430 and the Bluetooth chip, this proposed system is able to achieve a satisfactory sampling frequency of 2,000 Hz. The advantage of this system is the high sampling rate (2,000 Hz) than that of most commercial wireless EMG recording system (around 1,000 Hz or below) [18]. It is expected to achieve sufficient sampling rate to meet the high frequency range of sEMG. Combined with MSP430 and Bluetooth system, the proposed sEMG recording is stable, low cost, low power and user friendly. Bluetooth transmission is often interrupted, due to environmental electromagnetic wave interference or concrete structure blockage. This system can record sEMG data with its envelope being stored in a buffer. When the Bluetooth is suddenly interrupted and communication stopped, buffered data will be transmitted when the system is reconnected.

This proposed system was used for muscle fatigue examination under exercise training. The main requirement of exercise sEMG recording is system stability. This proposed system is useful for detecting sEMG during elliptical trainer exercises. The wireless function provides more application possibilities than traditional sEMG recording systems, such as dynamic monitoring of specific muscle disorder patients, and for health care of elderly patients.

The well-known phenomenon of decreasing MF with increasing muscle fatigue is also demonstrated with this system. Females will suffer more muscle fatigue when exercising. This is also shown in the results with the MF regression slope for females being lower than for males. The gender difference is significant in each 10 minute session at the L4 and L6 levels. L6 level is the maximum voluntary contraction. Thus, its mean slope is smaller than the other levels, and there is a significant difference from the mean slope of L4 level, in Table 3. Although, for the correlation coefficient, the gender difference is also significant on levels L2 to L6 in Table 2, we could not find a relationship between the stability of sEMG and the force level, as shown in Table 3. This phenomenon is the same as in previous studies [13–15]. Moreover, in Table 3, we find that the mean slope of the L2 level is lower than that of the L4 level, and its standard deviation is larger than that of the L4 level. The reason for this is that the L2 level is the minimum voluntary contraction, and belongs to the beginning of the exercise. Thus, the degree of muscle fatigue is very light. In Figure 7, the MF distribution of the L2 level does not decrease gradually. This phenomenon has also been reported in previous studies [13,14].

This system can be a potential candidate for other sEMG related disorder recording. Homecare and rehabilitation are the two main applications. Older patient’s motion monitoring, such as those with Parkinson’s disease, will benefit from this wireless sEMG recording system.

## 6. Conclusions

A portable wireless muscle contraction activity monitoring system with monitoring of muscle fatigue has been developed. Standard isotonic and isometric muscle contraction can be effectively recorded. A high sampling frequency achieving 2 kHz of Bluetooth transmission is beneficial for walking and exercising. The standard muscle fatigue index, the regression coefficient, and slope of MF for three loading exercises were also examined. There is a significant difference in the regression slope between male and female subjects. This system could be useful for sEMG-related disorder recording, especially in the homecare and rehabilitation environments.

## Figures and Tables

**Table 1.** Subject characteristics.

**s**	**Male (n = 5)**	**Female (n = 5)**	**P value**
Age (years)	24 (2)	21 (2)	0.13
Weight (Kg)	78.6(10.8)	53.3(5.3)	0.0015 **
Height (cm)	176.5(6.8)	163.6(4.7)	0.0078 **
BMI (Kg-M^−2^)	25.0(3.9)	19.8(1.4)	0.02 *

Data are expressed as mean (standard deviation). BMI is Body Mass Index.

* P < 0.05;

** p < 0.01.

**Table 2.** Slope and Regression results of muscle fatigue examination for gender difference. Data are expressed as mean (standard deviation).

**Parameters**	**Levels**	**Male (n = 30)**	**Female (n = 30)**	**P value**

Slope (A)	L2	−0.0167 (0.0108)	−0.0160 (0.0175)	0.859
	L4	−0.0088 (0.0070)	−0.0161 (0.0113)	0.004 **
	L6	−0.0128 (0.0095)	−0.0257 (0.0175)	0.0008 ***
	All	−0.0170 (0.0121)	−0.0224 (0.0130)	0.098

Correlation coefficient (r)	L2	0.6079 (0.2366)	0.4192 (0.2774)	0.006 **
	L4	0.4912 (0.2259)	0.6383 (0.2196)	0.013 *
	L6	0.5080 (0.2220)	0.6393 (0.2729)	0.045 *
	All	0.8154 (0.1910)	0.8246 (0.1824)	0.849

* P < 0.05;

** p < 0.01;

*** p < 0.001.

**Table 3.** Slope and Regression result of muscle fatigue examination for all subjects. P value is test result of t-test. Data are expressed as mean (standard deviation).

**Levels**	**Slope (A)**	**p-value**	**Correlation coefficient (r)**	**p-value**

L2	−0.0164 (0.0144)	*vs.* L4 = 0.08 *vs.* L6 = 0.28	0.5136 (0.2727)	*vs.* L4 = 0.27 *vs.* L6 = 0.21
L4	−0.0125 (0.0100)	*vs.* L6 = 0.0047 **	0.5647 (0.2330)	*vs.* L6 = 0.84
L6	−0.0193 (0.0154)	N.A.	0.5737 (0.2554)	N.A.
ALL	−0.0197 (0.0128)	N.A.	0.8200 (0.1852)	N.A.

* P < 0.05;

** p < 0.01;

*** p < 0.001.
